# Potential metabolic and genetic interaction among viruses, methanogen and methanotrophic archaea, and their syntrophic partners

**DOI:** 10.1038/s43705-022-00135-2

**Published:** 2022-06-28

**Authors:** Long Wang, Yinzhao Wang, Xingyu Huang, Ruijie Ma, Jiangtao Li, Fengping Wang, Nianzhi Jiao, Rui Zhang

**Affiliations:** 1 State Key Laboratory of Marine Environmental Science, College of Ocean and Earth Sciences, Xiamen University, Xiamen, China; 2 Southern Marine Science and Engineering Guangdong Laboratory (Zhuhai), Zhuhai, China; 3 State Key Laboratory of Microbial Metabolism, School of Life Sciences and Biotechnology, Shanghai Jiao Tong University, Shanghai, China; 4 State Key Laboratory of Marine Geology, Tongji University, Shanghai, China

**Keywords:** Microbial ecology, Microbial ecology

## Abstract

The metabolism of methane in anoxic ecosystems is mainly mediated by methanogens and methane-oxidizing archaea (MMA), key players in global carbon cycling. Viruses are vital in regulating their host fate and ecological function. However, our knowledge about the distribution and diversity of MMA viruses and their interactions with hosts is rather limited. Here, by searching metagenomes containing *mcrA* (the gene coding for the α-subunit of methyl-coenzyme M reductase) from a wide variety of environments, 140 viral operational taxonomic units (vOTUs) that potentially infect methanogens or methane-oxidizing archaea were retrieved. Four MMA vOTUs (three infecting the order *Methanobacteriales* and one infecting the order *Methanococcales*) were predicted to cross-domain infect sulfate-reducing bacteria. By facilitating assimilatory sulfur reduction, MMA viruses may increase the fitness of their hosts in sulfate-depleted anoxic ecosystems and benefit from synthesis of the sulfur-containing amino acid cysteine. Moreover, cell-cell aggregation promoted by MMA viruses may be beneficial for both the viruses and their hosts by improving infectivity and environmental stress resistance, respectively. Our results suggest a potential role of viruses in the ecological and environmental adaptation of methanogens and methane-oxidizing archaea.

## Introduction

Methane is a potent greenhouse gas that can significantly influence the Earth’s climate [1], and therefore is a critical component in global carbon cycling. Biogenic methane production is mostly by methanogenic archaea through methanogenesis in anoxic environments [2]. Methane can be oxidized by anaerobic methane-oxidizing archaea (ANME) via a reversed-methanogenesis pathway, usually in anoxic sediments, which significantly reduces methane emission into the atmosphere [3–5]. Currently, pure cultured or enriched methanogens and methane-oxidizing archaea (MMA) are only found in eight orders of the *Euryarchaeota*, although emerging metagenomic evidence indicated that more phyla possibly involved in the anaerobic metabolism of methane, such as *Candidatus* Verstraetearchaeota, Korarchaeota, Nezhaarchaeota etc [6–8]. MMA usually survive in mutualistic ways [1]. Most of ANME rely on syntrophic interactions with sulfate-reducing bacteria (SRB), except for ANME-2d, or occasionally ANME-1 [9, 10]. Many methanogens also benefit, although not obligatory, from syntrophic bacteria, such as SRB from the phylum *Firmicutes*, carbohydrate-fermenting bacteria from the phylum *Chloroflexi*, and acetate-oxidizing bacteria from the class *Deltaproteobacteria* [11–14].

In most anoxic environments, viruses are the main biological controlling factors of indigenous microbial communities such as MMA [15]. However, our knowledge about viruses that can infect MMA is scarce. Currently, only few viruses that infect methanogens have been isolated, such as *Myoviridae* ΦFl (infects *Methanobacterium* sp.) [16], *Siphoviridae* ψM1 (infects *Methanothermobacter marburgensis* Marburg) [17], and *Tectiviridae* MetSV (infects *Methanosarcina mazei* Gö1) [18]. Generally, most cultured MMA viruses have been isolated from engineered ecosystems (e.g., anaerobic sludge, Supplemental Table 1). Using culture-independent methods, a virus that potentially infects the methanogen *Methanosarcina barkeri* Fusaro with high abundance was identified from hydrocarbon polluted sediment metagenomes [19]. Paul et al. recovered an ANME virus from deep subsurface virome coding for the diversity-generating retroelements system, which may enhance the genomic diversification of their hosts and confer additional selective advantages in the energy-limited environment [20]. Based on this limited information, we hypothesized that diverse and distinct viral groups are widely distribute in MMA inhabiting environments. Considering the close ecological relationships between MMA and their mutualistic partners, we further hypothesized that interactions exist between them and their viruses. Therefore, to achieve a better understanding of MMA viruses, we investigated metagenomes that may inhabit MMA from a variety of habitats and expanded knowledge of the diversity, distribution, life strategies, and possible ecological roles of MMA viruses.

## Results and discussion

To evaluate potential viral attack on MMA, we analyzed all MMA genomes in the NCBI RefSeq database (*n* = 301; Aug 5, 2020). More than 74% (86 of 116) of the complete MMA genomes contain at least one level 4 CRISPR and Cas cluster (CRISPRCasdb database, version Jan 21, 2021) and the percentage is higher than the average levels of all archaea (70%) and bacteria (36%) [21]. Furthermore, the proportion of MMA genomes with at least one provirus was 41.9% (126 of 301), higher than the average level (~30%) when considering all microbial genomes [22]. These findings indicate that MMAs are under severe threat from viral infection, whereas CRISPR Cas system is a vital method for virus defense.

**Fig. 1:**
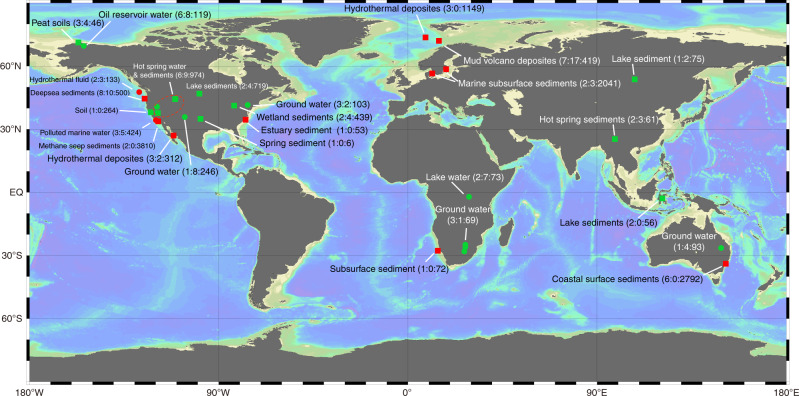
Geographic locations of metagenomic samples analyzed in this study. A simple description of ecosystems for each site are pointed to sites. The number of samples, number of MMA MAGs and viral contigs recovered from each site separated by colons were illustrated in parenthesis. Squares and circles indicate soil/sediment and water samples, respectively; red and green indicate samples from the ocean and land, respectively.

The gene coding for the α-subunit of methyl-coenzyme M reductase (*mcrA*), the key enzyme in both the methanogenesis and anaerobic methane-oxidizing pathways, is usually used as a marker gene for the detection and phylogenetic analysis of MMAs [8, 23]. Therefore, we analyzed 74 public *mcrA* gene-containing metagenomic datasets from diverse natural ecosystems, including marine and lake sediments, hot spring sediments, peatland soil, ground water, and hydrothermal vents (Fig. 1, Supplemental Table 2). The relative abundance of MMA averaged 4.6% across all samples and reached as high as 73.3% in a mud volcano sediment. *Methanosarcinales* was the dominant order of MMAs, followed by *Methanomicrobiales* and *Methanobacteriales* (Supplemental Fig. 1). A total of 2050 high quality metagenomic assembled genomes (MAGs) were recovered from the 74 samples. *Deltaproteobacteria* (*n* = 168), *Chloroflexi* (*n* = 163), *Bacteroides* (*n* = 148), and *Gammaproteobacteria* (*n* = 137) were the most widespread bacterial MAGs. Among the 565 archaeal MAGs, 82 affiliated to the eight typical MMA orders. Functional annotation revealed 12 more MAGs belonging to *Candidatus* Bathyarchaeota, Korarchaeota, and Verstraetearchaeota with methyl-coenzyme M reductase, which were considered as MMA as well (Supplemental Table 3). Among 94 MMA MAGs obtained in this study, 31 MAGs had at least one level 4 CRISPR and Cas cluster. The lower rate of MAGs with CRISPR Cas system compared with reference MMA genomes is probably because the presence of CRISPR spacers can influence the tetranucleotide frequency calculation and interfere binning processes [24].

### Viruses are widely distributed in MMA inhabiting environments

**Fig. 2:**
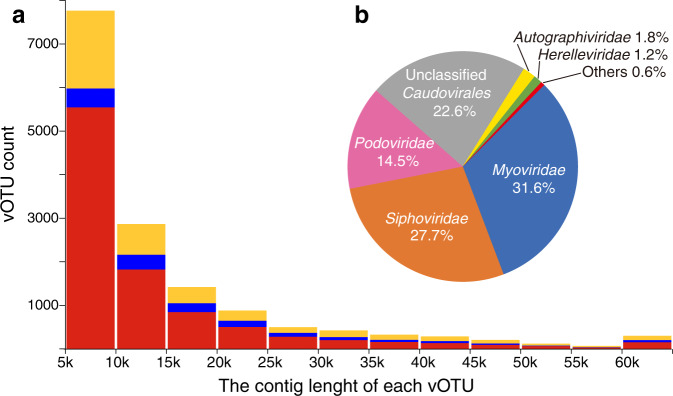
Quality and taxonomic diversity of viral operational taxonomic units (vOTUs) identified in this study. **a** Histogram of the contig lengths of vOTUs. Red indicates that the vOTUs were novel compared with the IMG/VR database; blue indicates that the vOTUs clustered with contigs in the IMG/VR database, whereas they were more complete; yellow indicates that the vOTUs were found in the IMG/VR database with equal or lower completeness. **b** Taxonomic affiliation of predicted vOTUs comparing with the NCBI viral reference database using the lowest common ancestor algorithm.

Using combined searches with the Earth’s virome protocol [25], VirSorter [26], and DeepVirFinder [27], 17,350 viral contigs were retrieved from the 75 datasets. By integrating the ~2.33 million viral sequences from the IMG/VR database (v3.0) [28], all viral contigs were clustered into approximate species level virus operational taxonomic units (vOTUs) at 95% average nucleotide identity (ANI), resulting 988,888 vOTUs in total. After excluding the vOTUs only composed of viral sequences from IMG/VR database, 15,048 vOTUs containing viral contigs identified in the present study were used for further analysis, in which 5714 vOTU representative sequences were >10 kb, with four contigs >200 kb, the latter conforming to the definition of giant viruses (Fig. 2a; Supplemental Table 4). As estimated by CheckV, 577 viruses were complete or with high quality [29]. Compared with the latest IMG/VR database, ~65% (9761) and 9.7% (1463) of the vOTUs identified in this study were novel, or of higher quality, respectively. Moreover, 59.3% of the vOTUs were only found in one of the 74 datasets and >90% of the vOTUs were distributed in <4 samples, suggesting a high diversity and endemic of the viruses within the studied environments (Supplemental Fig. 2a). On the other hand, 78.8% vOTUs could be assigned taxonomy, mostly as tailed viruses (*Caudovirales*), which was dominated by *Myoviridae* (31.6%), followed by *Siphoviridae* (27.7%) and *Podoviridae* (14.5%) (Fig. 2b). The distribution range of *Myoviridae* was significantly wider than *Siphoviridae* and *Podoviridae* (Mann–Whitney ranked *t*-test, *p* < 10^−4^; Supplemental Fig. 2b).

The lifestyle of viruses is a critical factor to evaluate their ecological significance. Lytic infection more likely indicates top-down control of the host community [30], whereas lysogenic infection usually regulates the metabolism of prokaryotic hosts [31]. In this study, both the presence of integrase genes annotated by the Pfam database and the integration of viral regions into host genomes predicted by VirSorter were used as signatures for a lysogenic viral lifestyle [32]. Among the 15,048 vOTUs identified in this study, a total of 978 vOTUs were considered as proviruses (Supplemental Table 4). Among the 577 high quality or complete vOTUs, at least 21.32% (123 of 577) had potential to enter the lysogenic cycle. Recently, a provirus (MFTV1) infecting hyperthermophilic *Methanocaldococcus fervens* AG 86 ^T^, which had been predicted using similar in silico methods with the present study [33], was successfully induced by low temperature stress [34].

### The MMA viruses are diverse and unique

Using a modified in silico host prediction pipeline [28], 141 vOTUs were predicted to infect methanogens or methane-oxidizing archaea, expanding by >40% of the current cultured or uncultured MMA viral diversity in public databases. The orders *Methanosarcinales* (94 vOTUs), *Methanobacteriales* (18 vOTUs), and *Methanomicrobiales* (9 vOTUs) were the most prevalent hosts of the predicted MMA viruses (Supplemental Table 5). Among the viruses of *Methanosarcinales*, 20 vOTUs were predicted to infect ANME-2. For the MMA viruses with taxonomic assignment, more vOTUs were classified as *Siphoviridae* (39%) than *Myoviridae* (26%). High relative abundance of MMA viruses can be observed, especially in methane seep and marine sediments (Supplemental Fig. 3). Each MMA virus was generally distributed in same or similar environments, although certain vOTUs were recovered from diverse ecosystems.

**Fig. 3:**
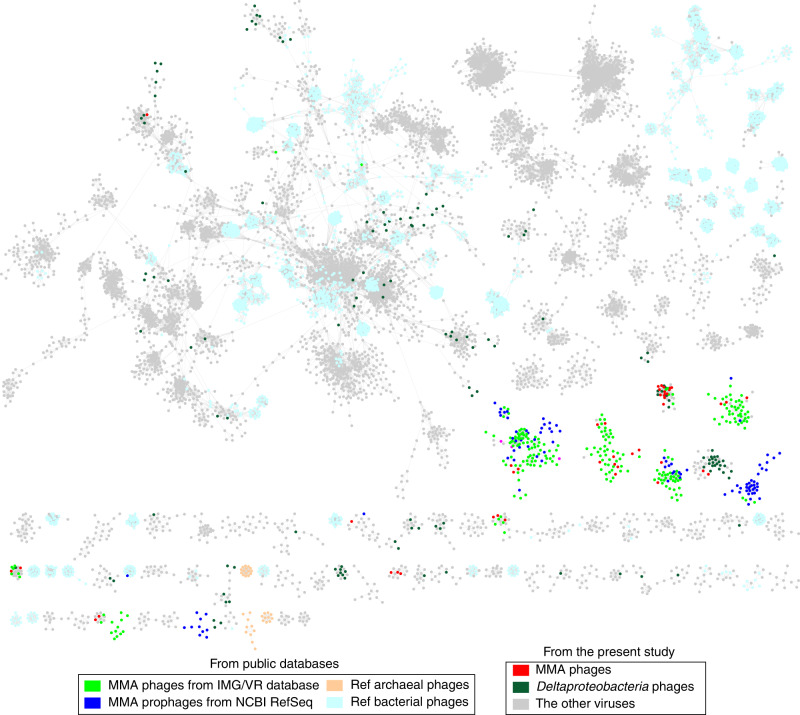
Relationship between vOTUs identified in this study and reference viral genomes. Gene-sharing network of viral sequences including all vOTUs from this study (*n* = 15,048), MMA virus vOTUs from the IMG/VR database (*n* = 349), provirus regions of MMA from the NCBI RefSeq database (*n* = 140), and RefSeq prokaryotic viruses (*n* = 3464). Nodes (circles) represent genomes and contigs, and shared edges (lines) indicate shared protein content. Only edges with a significance coefficient ≥10 are illustrated.

To investigate the genomic similarity of the predicted MMA viruses with publicly available sequences, a shared protein content-based network analysis [35] was performed with four datasets to produce genus-level viral clusters (VCs): (1) 3464 prokaryotic viral genomes, including 107 archaeal viruses and 3357 bacterial phages (RefSeq v99); (2) all 15,048 vOTUs mined in this study; (3) 140 provirus regions (Supplemental Table 6) identified from 301 MMA genomes (Supplemental Table 7) from the NCBI RefSeq database; and (4) 349 MMA vOTUs acquired from the IMG/VR database (v3.0). The gene-sharing network revealed that no MMA viruses/proviruses could form VCs with viral genome from RefSeq v99, except for two methanogens viral isolates (*Methanothermobacter* virus ψM100 and *Methanobacterium* virus ψM2) (Fig. 3), which reflected the uniqueness of the viruses infecting MMA. By contrast, the MMA viruses derived from the present study and the public databases formed cohesive clusters, suggesting that MMA viruses with various origins share similar core genomic characteristics. However, variation of the pan-genomic traits could be observed between the MMA viruses identified in this study, which are all from natural environments, and those from the IMG/VR database primarily identified from animal-associated (32.3%) or engineered (55.9%) ecosystems [28]. Among the 2640 protein clusters (PCs) encoded by MMA viruses from natural ecosystems (140 and 51 MMA viruses from the present study and the IMG/VR database, respectively), 43.2% (1140) of the PCs are unique (Supplemental Fig. 4). Furthermore, 46.0% of the PCs of MMA viruses from engineered and animal-associated ecosystems are distinct compared with those from the other environments. The lower nutrient concentrations and higher complexity of redox gradients in the investigated natural ecosystems [36, 37] may contribute to the differences in the viral genomic signatures. On the contrary, for the 64 shared PCs, they generally related to some core functions of viruses, such as structure (capsid, portal, and tail), replication (DNA methylase, DNA polymerase, integrase, and transposase), lysis (holin), packaging (terminase), and toxin-antitoxin systems.

**Fig. 4:**
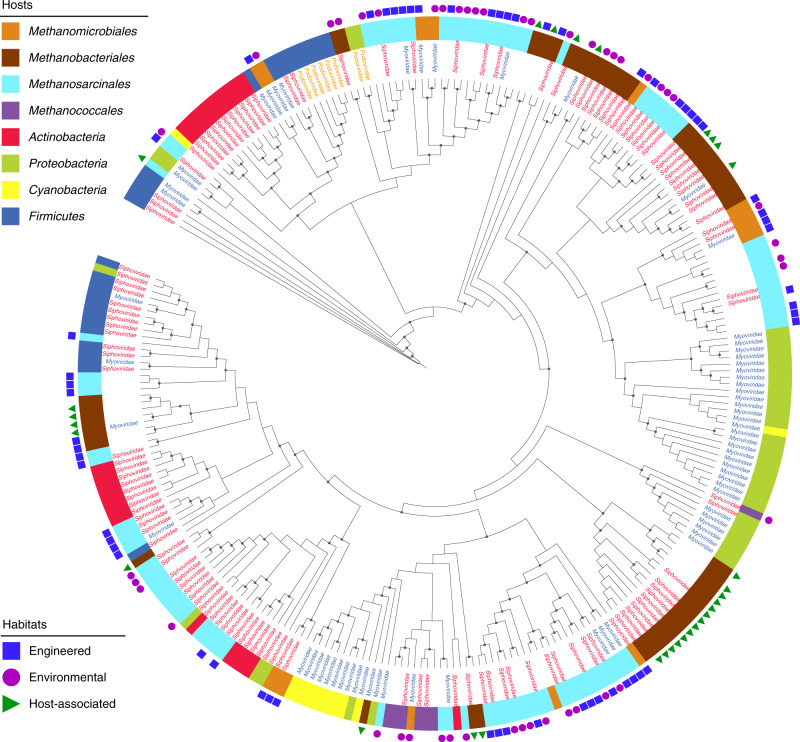
Phylogenetic comparison of MMA viruses. Maximum likelihood tree of terminase large subunit N-terminal domain (T4-like virus type) of MMA viruses and NCBI reference viruses. Branch length was ignored in this cladogram tree. Text labels indicated the taxonomy of the viruses; ring color indicated the hosts taxonomy of viruses; blue square, purple circle and green triangle indicated the viruses recovered from engineered, environmental and host-associated ecosystems, respectively. The gray circles on nodes indicate bootstrap values ≥90.

To investigate the phylogenetic variation between MMA viruses and NCBI reference viruses, terminase large subunit were used to construct a maximum likelihood tree (Fig. 4). Clear evolution variations could be observed of MMA viruses and other viruses, as well as the viruses infecting MMA of different orders. Furthermore, terminase large subunit encoded by MMA viruses clustered according to their taxonomy and inhabiting environments. The tetranucleotide frequencies of viruses infecting different orders of MMA were compared (Supplemental Fig. 5). Except for *Methanomassiliicoccales* and their viruses, *Methanomicrobiales*, *Methanobacteriales*, *Methanosarcinales*, and *Methanococcales* were illustrated similar tetranucleotide frequencies with their viruses. Moreover, similar tetranucleotide frequencies could be observed for viruses infecting MMA from same order, although their tetranucleotide frequency variations were higher than their hosts.

### MMA viruses may infect mutualistic *Deltaproteobacteria*

Many proteins encoded by MMA viruses were found having high homology with proteins from *Deltaproteobacteria*. To uncover the relationships between MMA viruses and *Deltaproteobacteria*, host prediction was conducted with 386 vOTUs that were identified as potentially infecting *Deltaproteobacteria* using the same host prediction pipelines (Supplemental Table 8). Interestingly, four vOTUs were predicted to infect both MMA and *Deltaproteobacteria* (Supplemental Fig. 6). Specifically, 11 and 13 proteins of vOTU SRR3715733_6188278_L48927 were best hits to MMA and *Deltaproteobacteria*, respectively, in a BLASTP search of the NCBI nr database. Gene cluster I, containing viral structure-related genes showed high similarity with MMA, whereas gene cluster II was homologous to *Desulfuromonadales* bacterium C00003093 (GCA_001751205.1). The recombination of the viral genome with MMA and *Deltaproteobacteria* implied a possible cross-domain infection. For vOTU SRR5214151_scaffold00034_L57801, the genomic region encoding tail- and capsid-related genes is homologous to a prophage integrated in a deltaproteobacterial genome (GCA_007280345.1), while the match between the viral protospacer and CRISPR spacer of *Methanobrevibacter olleyae* (GCF_001563245.1) indicates a previous infection of MMA. Moreover, the anti-recBCD protein 1 (*abc*1) encoded by this viral contig was proved able to inhibit RecBCD nuclease, a complex with central anti-phage functions in bacteria [38, 39]. Considering the *abc*1 gene is located adjacent to three type IV CRISPR-Cas protein-encoding genes (*csf2*, *csf3*, and *csf4*), we suspect a possible function of this gene island in viruses in resisting MMA CRISPR-Cas immune systems. A mechanism for resisting host CRISPR-Cas systems may be important for viruses to expand their host-range [40].

Emerging evidence derived from recent ecological and metagenomics studies indicates that viruses with broad host-range are far more prevalent than previously thought [41, 42], yet the underlying mechanism is still poorly understood. From the IMG/VR database v3.0, we found similar evidence of cross-domain infection by MMA viruses, which were also predicted to infect *Anaerolineales* (*Chloroflexi*), or *Syntrophorhabdus* and/or *Smithella* (*Deltaproteobacteria*) (Supplemental Fig. 6, Supplemental Table 9). As most MMA benefit from symbiotic heterotrophic bacteria, represented by *Deltaproteobacteria* and *Chloroflexi* [1], the close ecological relationships between MMA and their mutualistic partners are probably the reason why they are susceptible to the same viruses, or their viruses are similar in genetic characteristics. Viruses have long been deemed an important medium of horizontal gene transfer [43]. Viruses with broad host-range perhaps significantly contribute to gene transfer from bacteria to archaea, which has been implicated as a primary driver of archaeal metabolic innovation [44, 45], but the actual contribution awaits more experimental evidence.

### MMA viruses are associated with organosulfur metabolism

**Fig. 5:**
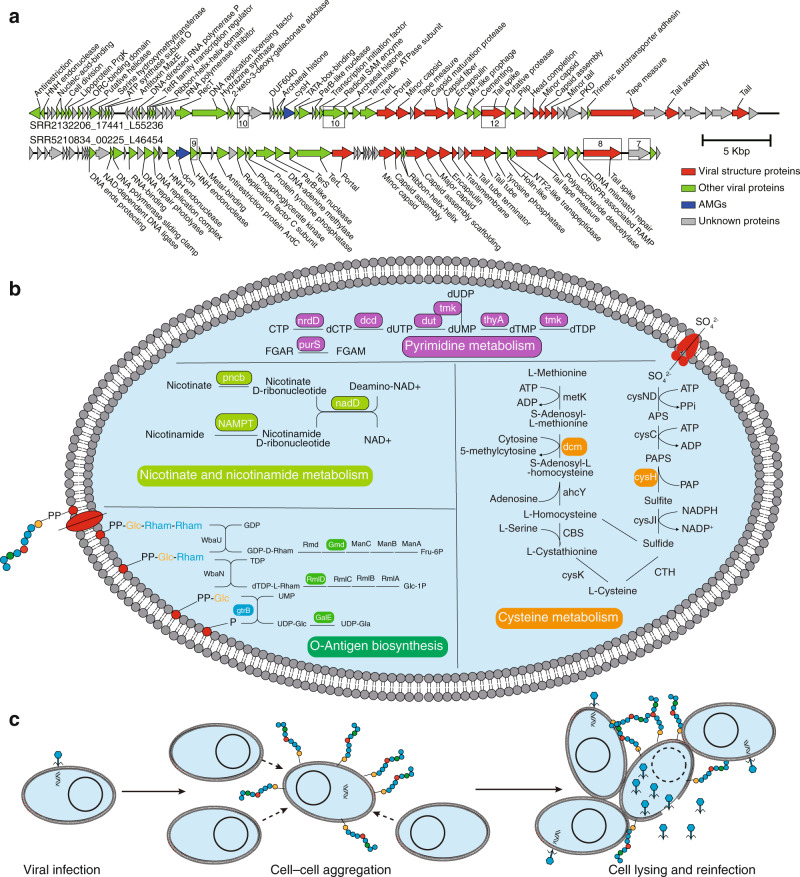
The potential influence of viruses on the metabolism of methanogens and methane-oxidizing archaea. **a** Genome maps of two representative vOTUs with phosphoadenosine phosphosulfate reductase (*cysH*) and DNA (cytosine-5)-methyltransferase (*dcm*), respectively. Red indicates proteins related to viral structure; green indicates proteins with viral origin; blue indicates *cysH* or *dcm*. The cysteine numbers of the top three cysteine abundant proteins are illustrated on the genome map. **b** Putative auxiliary metabolic genes (AMGs) identified in MMA viruses involved in pyrimidine, organosulfur, O-antigen, nicotinate and nicotinamide metabolism. The virus-encoded genes were highlighted in these pathways. **c** The infection cycle model of MMA viruses that encode genes related to O-antigen synthesis. These viruses enhance the synthesis of rhamnose in outer membrane lipopolysaccharide, which can facilitate cell-cell aggregation and make it easier for viruses to encounter potential hosts.

To investigate the mechanisms of MMA viruses interacting with their hosts and affecting biogeochemical cycles, virus-encoded putative auxiliary metabolic genes (AMGs) were predicted using DRAM-v, followed by manual curation, resulting in the identification of 44 putative AMGs (auxiliary score ≤ 3) related to various host metabolic functions, including carbohydrates, nucleotides, cofactors and vitamins, sulfur, and amino acid metabolisms (Supplemental Table 10). One of the most widespread and abundant putative AMGs was *cysH*, which encodes phosphoadenosine phosphosulfate reductase, a key enzyme of the assimilatory sulfate reduction pathway (Fig. 5a; Supplemental Table 10). *cysH* has frequently been reported to be carried by viruses from diverse anoxic ecosystems, including rumen [46], sulfidic mine tailings [47], stratified redoxcline [48], and cold seeps [32], but to be absent from oxic ecosystems [48]. As one of the limited electron acceptors in anoxic environments, sulfate plays vital roles in energy metabolism of microorganisms [49]. Meanwhile, sulfur is also an essential constituent of biomass [50], so the enhancement of sulfur uptake through assimilatory sulfate reduction for synthesis of sulfur-containing amino acids (methionine and cysteine) and other organic matters may help MMAs, as well as their viruses, to survive in anoxic ecosystems.

To explore the potential function of *cysH* in viruses, the frequency of cysteine and methionine codons was compared between viruses with and without a *cysH* gene identified in this study. Significant higher cysteine frequency was observed for the viruses containing *cysH*. However, the methionine frequency was not influenced by the presence of *cysH* (Supplemental Fig. 7). The widespread presence of DNA (cytosine-5)-methyltransferase (*dcm*) in viral genomes, which catalyzes the degradation of methionine, may explain this phenomenon. There were 154 vOTUs that contained the *dcm* gene, seven of which were predicted to infect MMA with one MMA virus carried both *cysH* and *dcm* genes. We also found that the viruses with the *dcm* gene had a higher frequency of codons for cysteine (but not methionine) in their genomes (Supplemental Fig. 7). The *dcm* gene from viruses may enhance the degradation of methionine to redirect organic sulfur to enter the cysteine biosynthesis pathway, although a potential function of *dcm* in helping viruses to protect DNA from being cut by native methylation-sensitive restriction enzymes has been reported [51]. Overall, we propose two strategies used by viruses in anoxic ecosystems to synthesize more cysteine, which is the only amino acid that can form disulfide bonds that stabilize viral protein structure [52]. Viruses enhance the assimilatory sulfate reduction pathway, and/or reuse the sulfur in methionine or other organosulfur from the host cell to synthesize cysteine (Fig. 5b).

To help uncover the function of cysteine in viruses, the two MMA viral genomes separately containing *cysH* and *dcm* genes were studied in detail (Fig. 5a). In both viruses, the tail spike protein was among the most cysteine-rich proteins. Tail spikes are responsible for attachment to cells, and frequently recognize and hydrolytically cleave bacterial cell surface polysaccharides [53]. The enrichment with cysteine of the tail spike may play a critical role in the disruption of the host membrane via specific and non-specific electrostatic and hydrophobic interactions with cell surface groups [54, 55]. Radical S-adenosyl-L-methionine (SAM) enzyme, which includes a vitamin B_12_-binding domain and a [4Fe–4 S] cluster, also has 10 cysteine and initiate diverse sets of radical reactions [56]. The three cysteine residues of the CX_3_CX_2_C motif coordinate three of the four irons of the [4Fe–4 S] cluster at the active site of the enzyme [56]. A huge virus identified in this study encoded 15 radical SAM enzymes (Supplemental Fig. 8), indicating a critical function of this gene for viruses in anoxic ecosystems. Overall, cysteine may be important for both the activity of enzymes and entry into host cells for viruses.

### MMA viruses adapt to their hosts and inhabiting ecosystems

The MMA viruses identified in this study encoded genes involved in the biosynthesis of O-antigen (including UDP-glucose 4-epimerase, GDP-mannose 4,6 dehydratase, and dTDP-4-dehydrorhamnose reductase) and other carbohydrate glycosyltransferases (GT2, GT4, and GT9) (Supplemental Fig. 9). The O-antigen is an important component of the outer membrane, but flexible and highly variable even among the closest of relatives [57–59]. Temperate phages from the gammaproteobacterial *Shigella flexneri* code for factors responsible for serotype conversion, which can protect *S. flexneri* from immune response directed against the O-antigen component of the outer membrane lipopolysaccharide (LPS) [60]. The O-antigen genes encoded by MMA viruses may enrich rhamnose and galactose in the LPS of their hosts by converting GDP-D-mannose to GDP-D-rhamnose, dTDP-4-dehydro-β-L-rhamnose to dTDP-L-rhamnose, and UDP-D-glucose to UDP-D-galactose. Rhamnose-rich O-antigen can contribute to surface adhesion and cell-cell aggregation [61], which is a strategy to protect microorganisms from environmental stresses [62, 63]. On the other hand, as the burst size of MMA viruses is generally small [17, 34], the aggregated cells may make it easier for viruses to encounter potential hosts (Fig. 5c). As well as the synthesis of O-antigen, the bactoprenol glucosyl transferase gene (*gtrB*) affiliated to GT2 encoded by MMA viruses catalyze the transfer of glucosyl from UDP-glucose to bactoprenol [64]. Moreover, research has revealed that the modification of host O-antigen and LPS by viruses (typically proviruses) probably acts as a protective mechanism to exclude other viruses from infecting by altering viral adsorption sites [65, 66]. Additionally, viruses may help with host defensive systems. A total of 12 MMA viruses identified both in this study and the IMG/VR database containing genes coding for non-toxic nonhemagglutinin type C, which in proviruses of *Clostridium botulinum*, protects against pH-mediated botulinum neurotoxin type C (BoNT/C) inactivation [67]. MMA viruses also contain diverse genes associated with the purine and pyrimidine metabolism pathways, to shift host metabolism toward nucleotide biosynthesis as an adaptation to viral replication [68]. Previous research found that viruses encoded various carbohydrate-active enzymes affiliated to glycoside hydrolases to augment the breakdown of complex carbohydrates to increase energy production and boost viral replication [32, 69–71]. However, the putative AMGs encoded by MMA viruses rarely related to organic matter degradation, consistent with the metabolism of methanogens and ANME, which barely use carbohydrates as carbon and energy sources.

## Conclusions

Together, we observed the high diversity and novelty of viruses from various natural environments that potentially inhabit MMA. Distinct genome signatures, wide distribution, and high abundance were observed for the viruses infecting methanogens or methane-oxidizing archaea. Several MMA viruses were predicted to be able to infect mutualistic sulfate-reducing bacteria such as *Deltaproteobacteria* and *Chloroflexi*. As adaptations to their inhabiting environment, complex strategies were proposed of MMA viruses to interact with their hosts, such as enhancing assimilatory sulfate reduction to synthesize organosulfur, protecting the defense systems of their hosts, and facilitating cell-cell aggregation to resist environmental stresses. Although intensive laboratorial or in situ experiments are needed to validate these results, the present study may expand our view of MMA viruses and provide clues to the survival strategies of viruses in anoxic environments.

## Materials and methods

### Data acquirement

Using a previously reported *mcrA* protein database (*n* = 153) containing sequences from the known methanogens and ANMEs [8], predicted protein sequences of high-throughput metagenomes were queried to identify metagenomic datasets containing MCR-based alkane metabolism-related genes, using Diamond [72] version 0.8.28.90 (identity ≥ 0.3, coverage ≥ 0.75, *e* value ≤ 1 × 10^–20^). A total of 74 genomic datasets were acquired and download from the NCBI Sequence Read Archive public database (Fig. 1; Supplemental Table 2). Raw reads were trimmed using the Sickle algorithm version 1.33 followed by the assembly conducted using MEGAHIT version 1.0.6-hotfix1 with default parameter. Moreover, to identified high degree of confidence CRISPR spacers and proviruses of MMA, 116 complete and 185 draft genomes of methanogens or ANMEs were download from NCBI reference sequence database (RefSeq). All biological sequence data was downloaded using NCBI datasets command line tools version 10.5.1 (https://www.ncbi.nlm.nih.gov/datasets).

### Prokaryotic community, genomic binning, and taxonomic annotation

To explore the prokaryotic composition of each sample, prokaryotic 16S rRNA miTags were extracted from quality-controlled reads using SortMeRNA [73] version 4.2.0 with default parameters. All 16S rRNA miTags were queried to the kraken2 database (pre-built 16S rRNA gene database of SILVA v138) using lowest common ancestor algorithm [74], and then adjusted using Bracken [75]. MetaPhlAn3 was used to evaluate the potential eukaryotic contamination with default parameters [76], which revealed that the eukaryotic reads account for <0.1% of all clean reads for all samples. A hybrid binning approach was employed to cluster metagenomic contigs for each sample. Before the binning, sequencing reads were mapped to a corresponding metagenome by Bowtie2 version 2.3.4 and SAMtools version 1.6 to calculate average sequencing depth for each contig [77, 78]. Then the generated depth profile and metagenomic contigs (≥2 kb) were input into MaxBin2 for a first binning with default parameters [79]. Resultant contig groups were individually imported into MetaBAT2 for a second binning with default parameters [80]. Finally, CheckM was employed to estimate quality of the genome bins, which with contamination ≤10% and completeness ≥50% were retained for subsequent analysis [81]. Since metagenomes were assembled and binned separately, resulting redundant MAGs, which were dereplicated at 99% ANI using dRep v2.6.2 (parameters: -comp 50 -con 10 -sa 0.99) [82]. The phylogenetic affiliations of all non-redundant MAGs were analyzed using the GTDB-Tk genome-based taxonomy (GTDB-Tk version 1.1.1 with GTDB version 89) [83].

### Recover and deduplicate viral contigs

Contigs ≥5 kb of all metagenomic samples were pulled for viral contig recovery by stepwise method according to the confidence of prediction strategy. (1) The virus detection pipelines of Earth’s virome [25] resulted in 9892 viral contigs; (2) 6264 more contigs were sorted as virus/provirus category 1 or 2 by VirSorter [26]; (3) Among the category 3 virus/provirus sequences, 1088 were identified as viruses using DeepVirFinder (score ≥ 0.9 and *p* < 0.05) [27]. For the 301 MMA genomes, proviruses were also predicted using VirSorter (categories 1 and 2) [22]. After merging the viral contigs identified from *mcr*A-containing metagenomes, provirus regions from 301 MMA genomes, and all viral contigs of IMG/VR database (v3.0) [28], species-rank virus groups “vOTUs” were clustered based on pairwise ANI at the thresholds of 95% identity over 85% alignment fraction (relative to the shorter sequence) using CheckV code [29], and the longest contig of each vOTU were kept as representatives for further analysis. The completeness of viral contigs was then estimated using the CheckV pipeline (end_to_end program) [29]. VirSorter2 version 2.1 was also used to evaluate the prediction of viruses according to their standard operating procedure with manual curation and generate “affi-contigs.tab” files needed by DRAMv to identify AMGs with parameters --prep-for-dramv and --provirus-off [84].

### Viral taxonomic assignment and distribution profiles

Two complementary approaches were used for taxonomic classification. Firstly, vConTACT2 was conducted using default parameters resulting in only 1.26% vOTUs (*n* = 192) taxonomically annotated. Secondly, all vOTU representatives were sorted using CAT version 5.0.4 against the NCBI Viral RefSeq proteins v207 setting default options except “--evalue 1e-5” with 78.8% vOTUs acquiring taxonomic assignment. By comparing the two methods, high consistent rate (97.8%) of the vOTUs classified by both methods at family level can be observed. The relative abundances of vOTUs were quantified using reads per kilobase per million mapped reads, which calculated by mapping the original trimmed reads to viral contigs using BBMap version 38.87 with default parameters (https://github.com/BioInfoTools/BBMap). If the percentage of vOTU contig covered by reads was ≥85%, this vOTU was considered being present in this sample.

### Methanogens and methane-oxidizing archaea viral prediction

From the 15,048 vOTUs identified in this study, the viruses possibly infecting methanogens or methanotrophic archaea were predicted using in silico methods based on previous reports [69, 85, 86]. The clustered regularly interspaced short palindromic repeat (CRISPR) spacers and their associated Cas proteins were searched using CRISPRCasFinder from the 301 MMA genomes and 94 MMA MAGs binned in the present study [87]. The searching resulted CRISPR arrays were further sorted into four evidence-levels (level 1–4), which with a higher evidence-level indicating a higher likelihood being a true-positive array [87]. *Search_oligodb* function of Usearch (v11.0.667) was used to compare all predicted viral sequences to the database of MMA CRISPR spacers (https://drive5.com/usearch). For each pair of viral sequence and putative host genome, a valid matching was confirmed when at least one hit had ≤1 mismatch over the entire spacer length. Similarly, the tRNA genes of vOTUs, MMA genomes and MAGs were identified by tRNAscan-SE (v2.0.3) setting the parameters “-G” [88]. Blastn was used to align viral encoded tRNAs to MMA derived sequences, with complete matches as confidential links between viruses and hosts. A virus meeting the following criterions was linked to MMA: (1) viral contigs matched to evidence level 4 CRISPR spacers (22 virus-host links were identified); (2) identical tRNAs predicted from vOTUs and MMA genomes (one virus-host link was identified); (3) viral contigs sorted as MMA or with at least 5 ORFs sorted as MMA by CAT against the NCBI non-redundant protein database (nr; version 2020.03.04) (80 virus-host links were identified); (4) viral contigs binned into a MMA MAG with at least one ORF sorted as MMA (18 virus-host links were identified). Moreover, same strategy was conducted to predict the phages of Deltaproteobacterial SRB.

### Gene-sharing based network analysis

To investigate the relationship between MMA viruses and publicly available viral sequences, vConTACT2 was used to construct a gene-sharing network, including all vOTUs acquired in the present study (*n* = 15,048), provirus regions identified from 301 MMA genomes from NCBI RefSeq database (*n* = 140), MMA viruses identified by IMG/VR database v3.0 (*n* = 349), and prokaryotic viral RefSeq v99 integrated in vConTACT2 (*n* = 3,464). All above sequences were pooled to call ORFs using Prodigal (parameters: -m, -p meta) [89], and the resulting protein sequences were clustered using vConTACT2 with default parameters [35]. The resulting network was visualized in Cytoscape v3.8.2 using edge-weighted spring-embedded mode [90]. Only the interactions between viruses with a score ≥10 were illustrated in network.

### Functional annotations of viral sequences

All ORFs of 15,152 vOTUs called by prodigal were functionally annotated against KEGG database (release 95.0) and Pfam database (release 33.0) using KofamScan version 1.2.0 (*E* value < 10^−5^) [91] and Pfamscan (-as) [92], respectively. For the MMA viruses, the annotation was further conducted using DRAMv [93] version 1.2.0 with AMGs predicted (--max_auxiliary_score 3). All putative AMGs were manual curated by checking the upstream and downstream genes following recent protocol [94]. Genes related to nucleotide metabolism were excluded because of their widespread in viral genomes. The ORFs of viral contigs illustrated in Fig. 5 and Supplemental Fig. 6 were compared with PDB protein data bank using the online service of HHpred [95]. The protein fold recognition of viral encoded O-antigen genes were modelled using PHYRE2 to confirm and further resolve functional predictions [96]. All O-antigen gene structures modelled by PHYRE2 had 100% confidence scores and >80% coverage. The viral genome maps were visualized using Easyfig version 2.2.5 [97].

### Phylogenetic and tetranucleotide analyses of MMA viruses

All proteins of MMA viruses/proviruses used in the network analysis with a terminase large subunit N-terminal domain (T4-like virus type) were queried to phylogenetic analysis. Protein sequences were aligned with MAFFT (–localpair – maxiterate 1000) [98] and then adjusted with trimAl (-automated1) [99]. Maximum likelihood tree was built using IQ-Tree v2.0.3 with model auto-detected (LG + G) and an ultrafast bootstrap of maximum iteration of 1000 [100] and visualized using Interactive Tree Of Life (iTOL) with branch length ignored [101]. Tetranucleotide frequencies of MMA and their viruses were calculated, clustered, and visualized using Emergent Self-Organizing Maps [102]. The correlation coefficients of the tetranucleotide frequencies of all MMA viruses were calculated using Python package pyani. The pairwise comparison of viruses infecting the five orders of MMA were conducted using ANOSIM.

## Supplementary information

Supplementary information

Supplemental Tables 1-9

## Data Availability

Metagenomic data are available in the NCBI Sequence Read Archive (https://www.ncbi.nlm.nih.gov/sra) database and detailed in Supplemental Table 2. All other data produced in the present study are all available in Supplemental materials.
