# Supplementary material for: Potential metabolic and genetic interaction among viruses, methanogen and methanotrophic archaea, and their syntrophic partners
Source: ISME Commun. 2022 Jun 28;2:50. doi: 10.1038/s43705-022-00135-2 (PMC9723712; doi:10.1038/s43705-022-00135-2)

Supplemental Information for

**Potential metabolic and genetic interaction among viruses, methanogen and methanotrophic archaea, and their syntrophic partners**

**Long Wang^1,2^, Yinzhao Wang^3^, Xingyu Huang^1^, Ruijie Ma^1^, Jiangtao Li^4^, Fengping Wang^3^, Nianzhi Jiao^1^, Rui Zhang^1,2^***

^1^ State Key Laboratory of Marine Environmental Science, College of Ocean and Earth Sciences, Xiamen University, Xiamen, China.

^2^ Southern Marine Science and Engineering Guangdong Laboratory (Zhuhai), Zhuhai, China.

^3^ State Key Laboratory of Microbial Metabolism, School of Life Sciences and Biotechnology, Shanghai Jiao Tong University, Shanghai, China.

^4^ State Key Laboratory of Marine Geology, Tongji University, Shanghai, China.

*** For correspondence E-mail: ruizhang@xmu.edu.cn; Tel: 86-592-2880152; Fax: 86-592-2185375.**

**Supplemental Fig. 1** The microbial community structures in 74 samples based on 16S rRNA gene relative abundance. (a) The heatmap of the prokaryotic communities at phylum level, except for the phylum Proteobacteria at class level. LOG10 transform of relative abundance was conducted to illustrate more details of low coverage clades. The left and top trees indicated the relationship among taxon and samples, respectively. (b) The relative abundances of the seven orders related to methanogen and methane-oxidizing archaea (MMA) except the order ‘Candidatus Methanophagales’ (anaerobic methanotroph ANME-1).


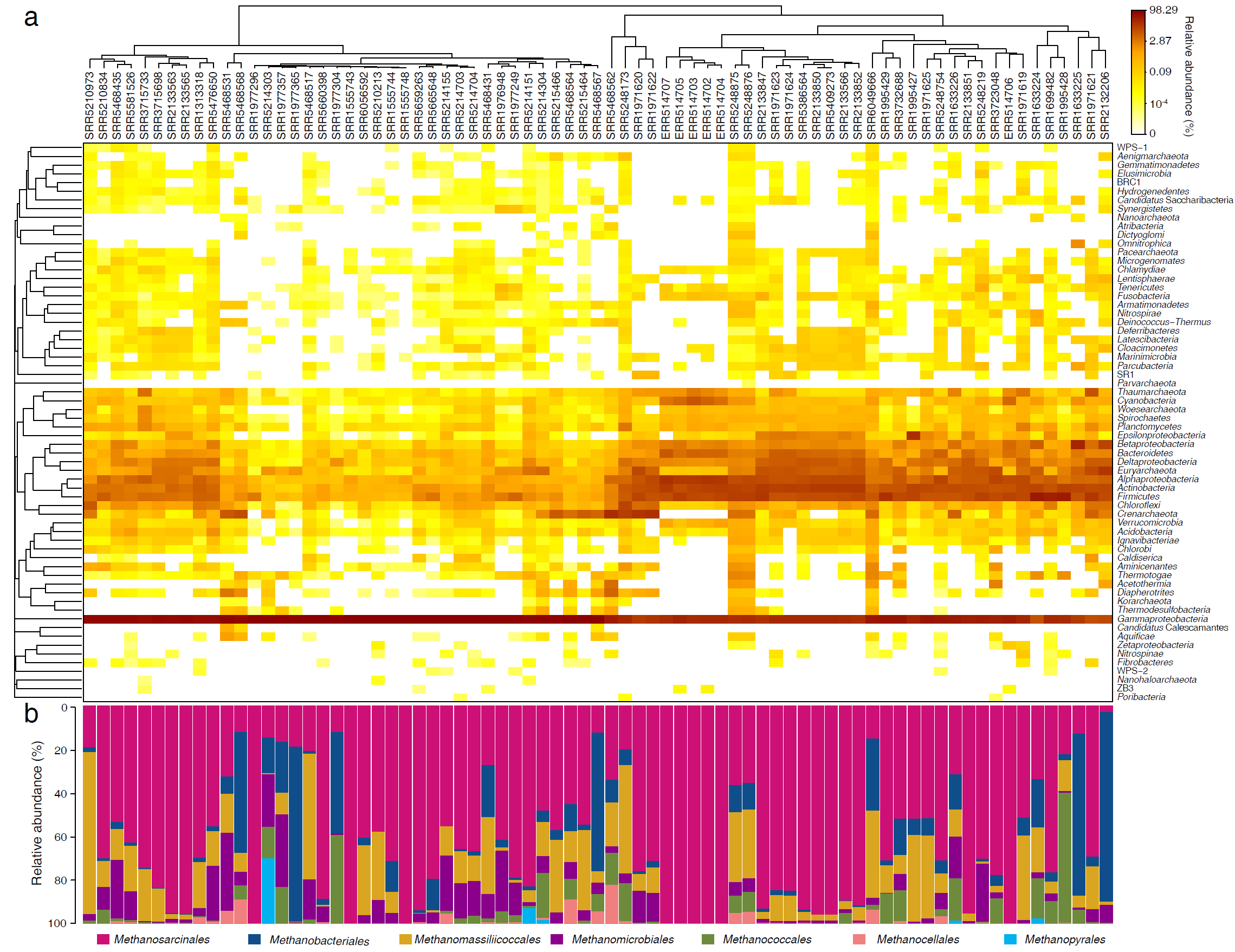


**Supplemental Fig. 2** Distribution pattern of vOTUs detected in this study. (a) The individual and cumulative abundance of vOTUs. Blue and orange circles indicate the viruses infecting MMA and Deltaproteobacteria, respectively. Gray rings indicate viruses infecting other hosts or unknown hosts. The column charts compare the distribution range of different viral taxonomies and hosts (b).


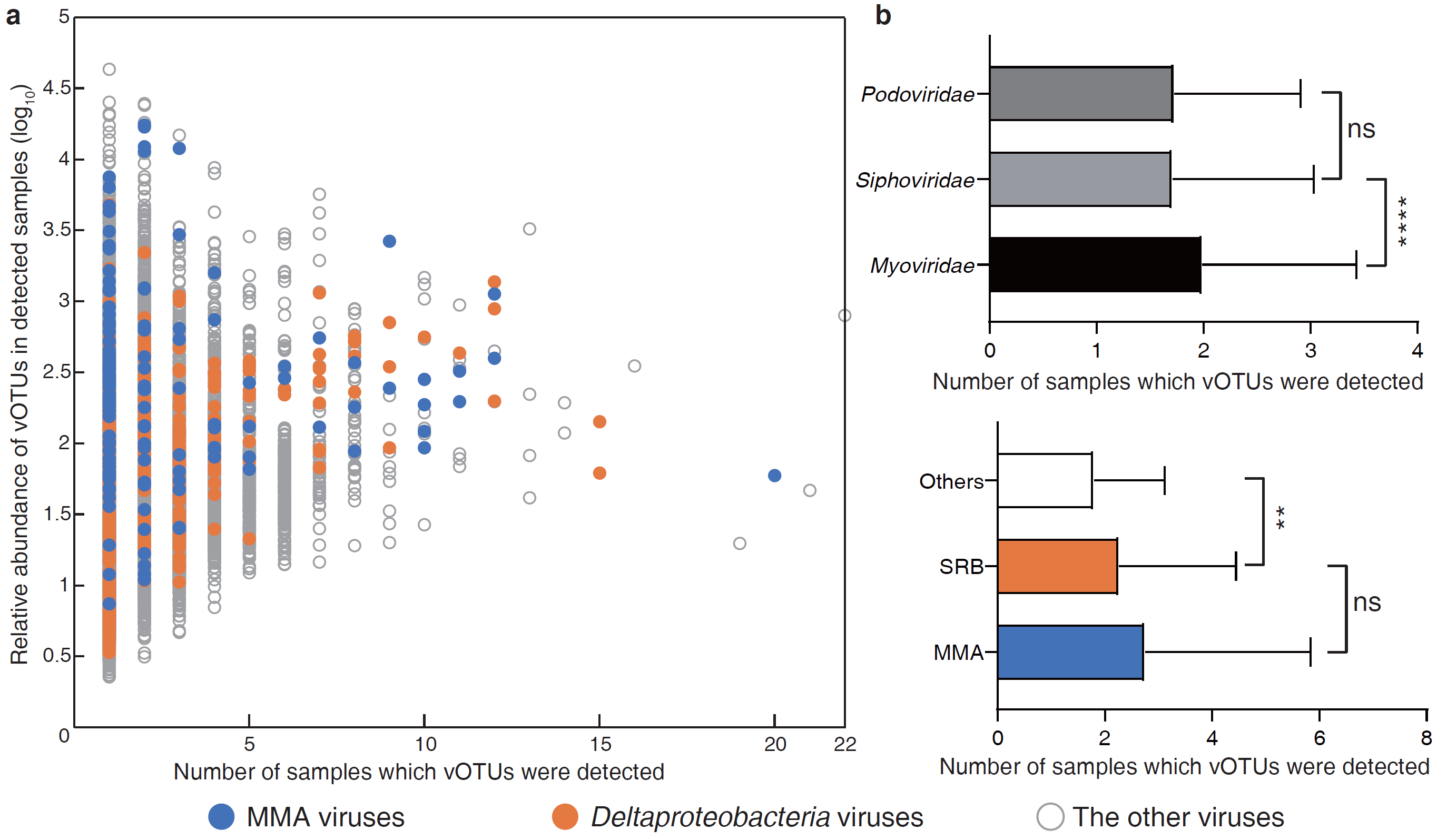


**Supplemental Fig. 3** The heatmap illustrated the reads coverage of the MMA viruses that identified in > 2 samples. LOG10 transform of reads coverage was conducted to illustrate more details of low coverage viruses. Rectangles with different colors indicated the predicted hosts of each vOTU; colored circles indicated the types of ecosystems that vOTUs distributed.


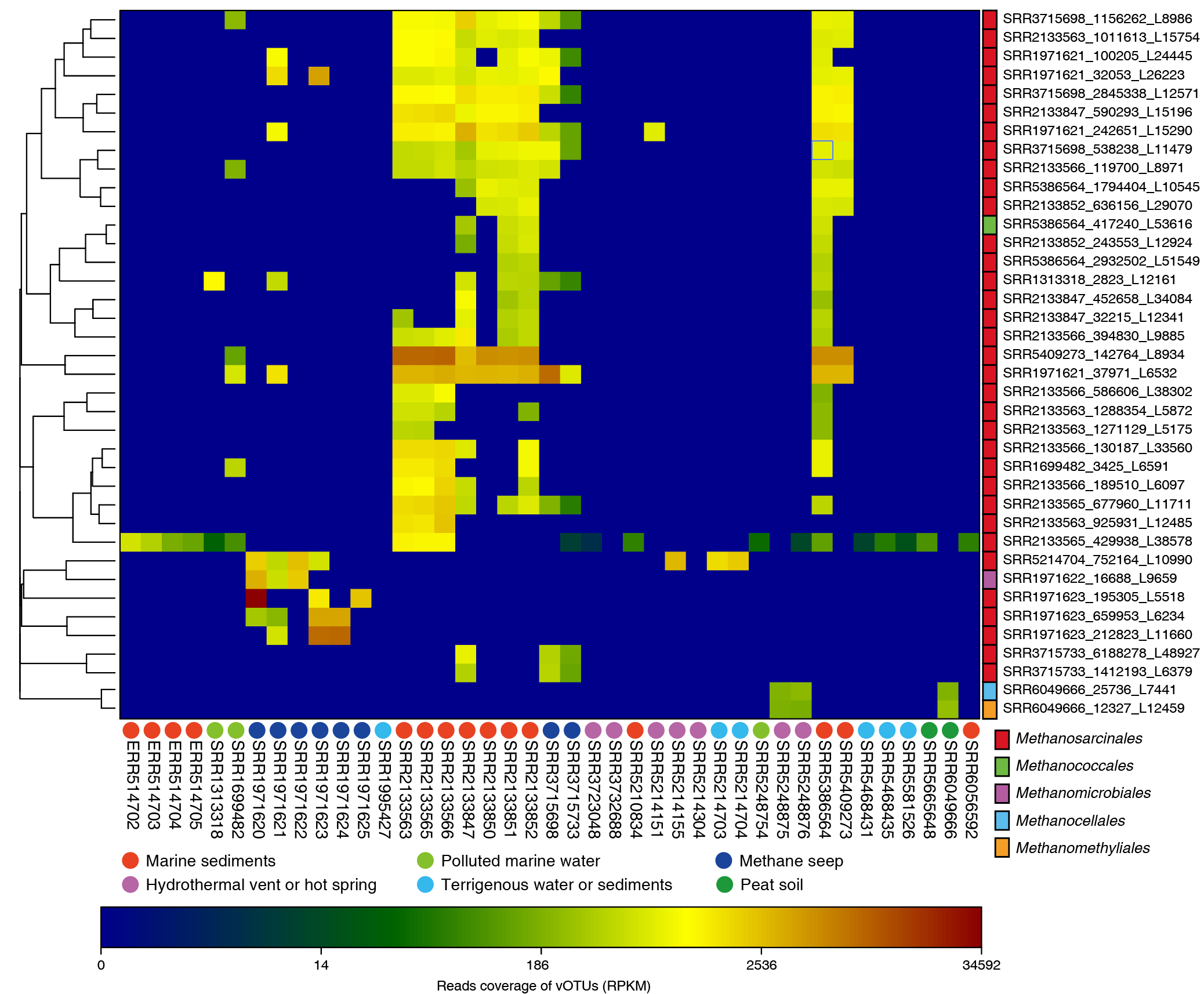


**Supplemental Fig. 4** Venn diagram of shared protein clusters (PCs) of MMA viruses retrieved from this study, IMG/VR database, MMA proviruses and archaeal viruses from RefSeq database.


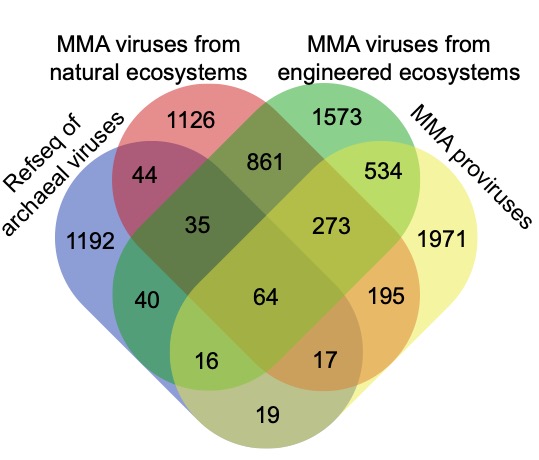


**Supplemental Fig. 5** Tetranucleotide frequencies comparison of MMA viruses. (a) High dimensional dataspace of tetranucleotide frequencies of representative MMA MAGs and all MMA viruses. Viruses were colored based on the taxonomy affiliations of their hosts. Each MAG was illustrated by different colors. More information of the MAGs can be found in Supplemental Table 3. (b) The pairwise comparison of viruses infecting the five orders of MMA. R values of ANOSIM analysis of each pair are illustrated.


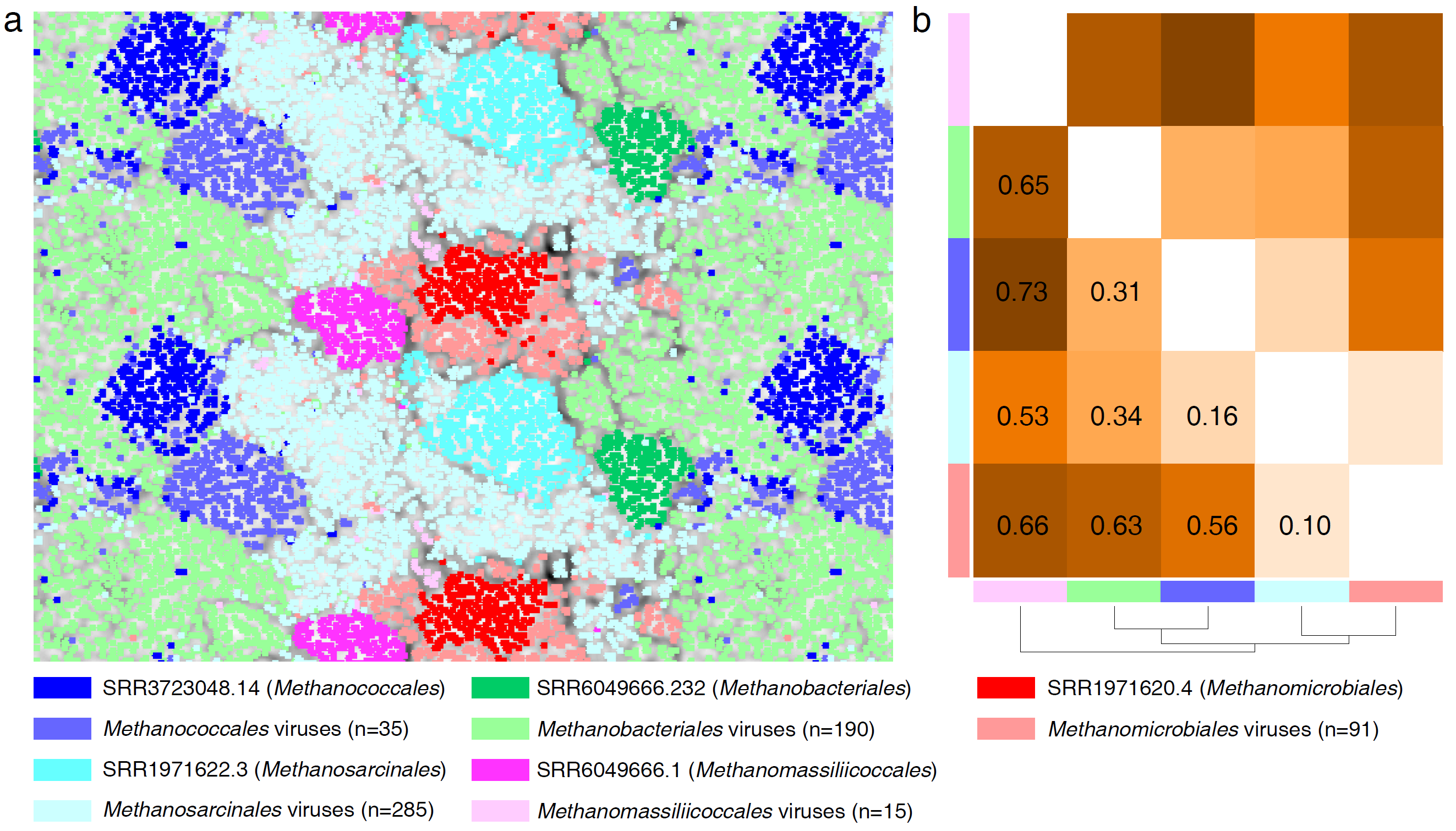


**Supplemental Fig. 6** Four vOTUs from this study and one vOTU from IMG/VR database that were predicted to infect both MMA and Deltaproteobacteria. Red and blue indicated that the genes were taxonomically assigned as MMA and Deltaproteobacteria, respectively. Arrows indicated the viral sequences were matched to the CRISPR spacers of MMA.


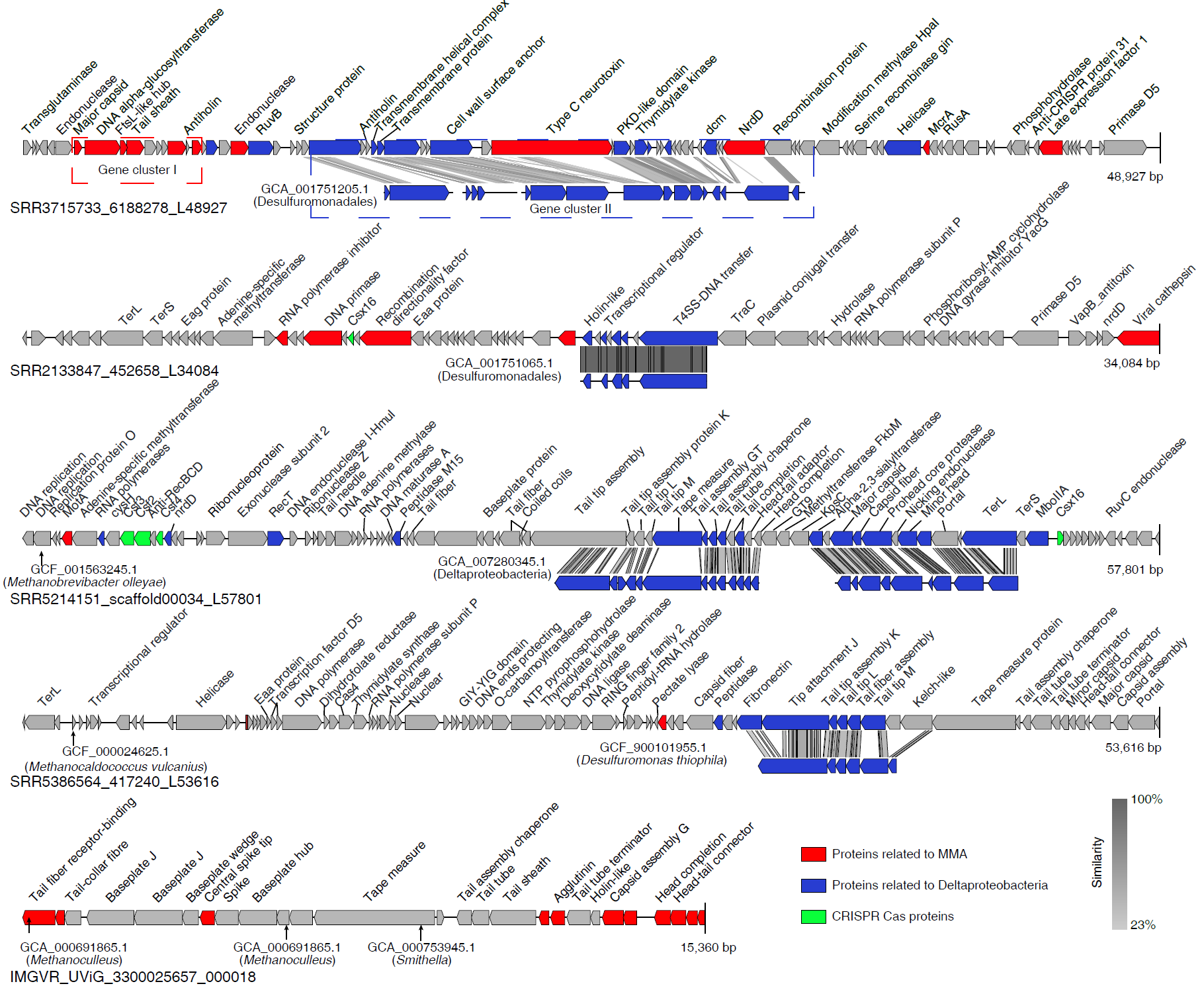


**Supplemental Fig. 7** The Mann-Whitney *t*-test was used to compare the cysteine frequency among viruses with the *cysH* gene, with the *dcm* gene, without *cysH* and *dcm* genes, and in all genomes of RefSeq v99.


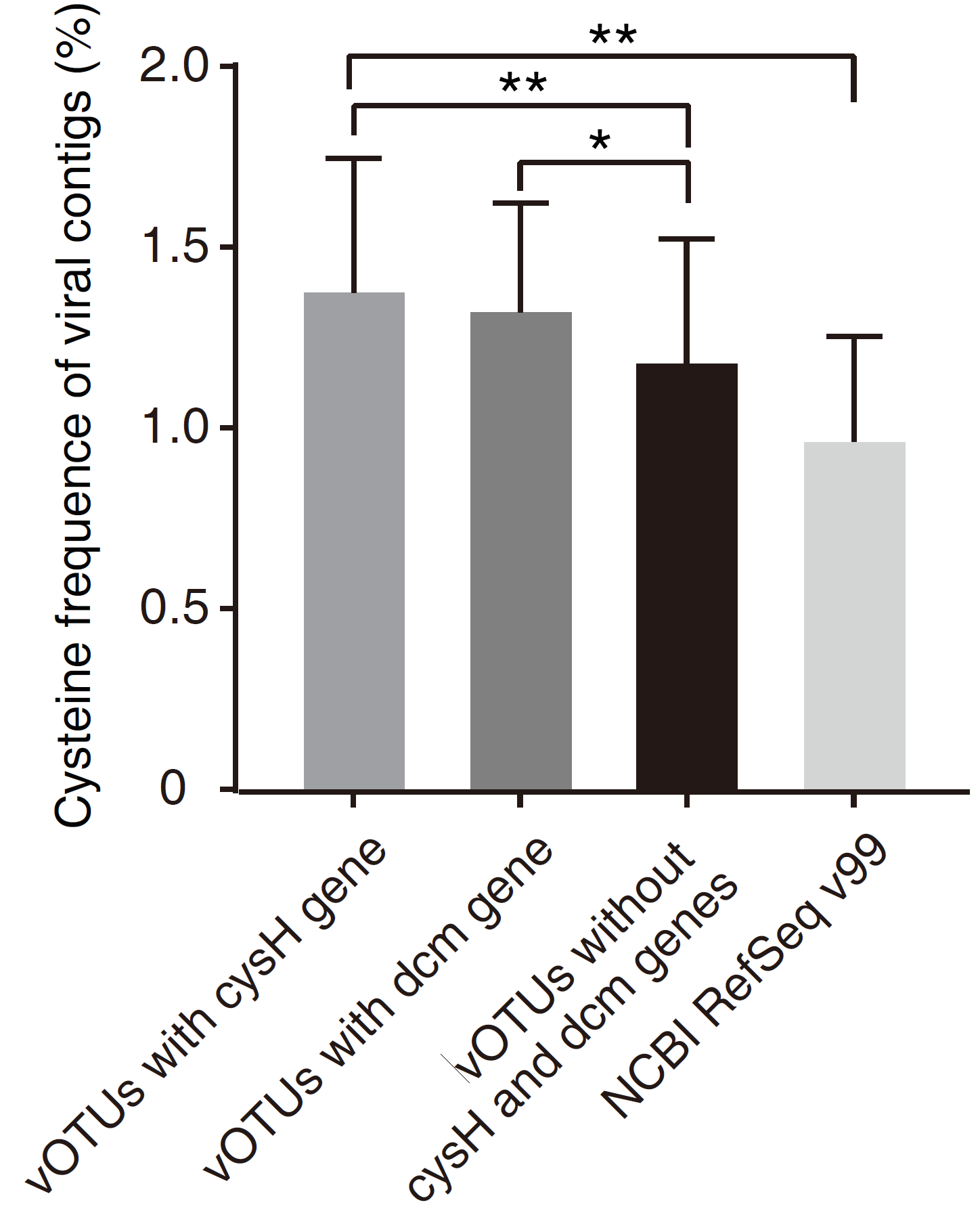


**Supplemental Fig. 8** The genome of the huge virus ERR514705_488141_L254223 encodes 15 radical SAM enzyme genes, which indicated by red. Blue indicates proteins related to virus structure; light blue indicates other viral proteins; brown indicates tRNA genes.


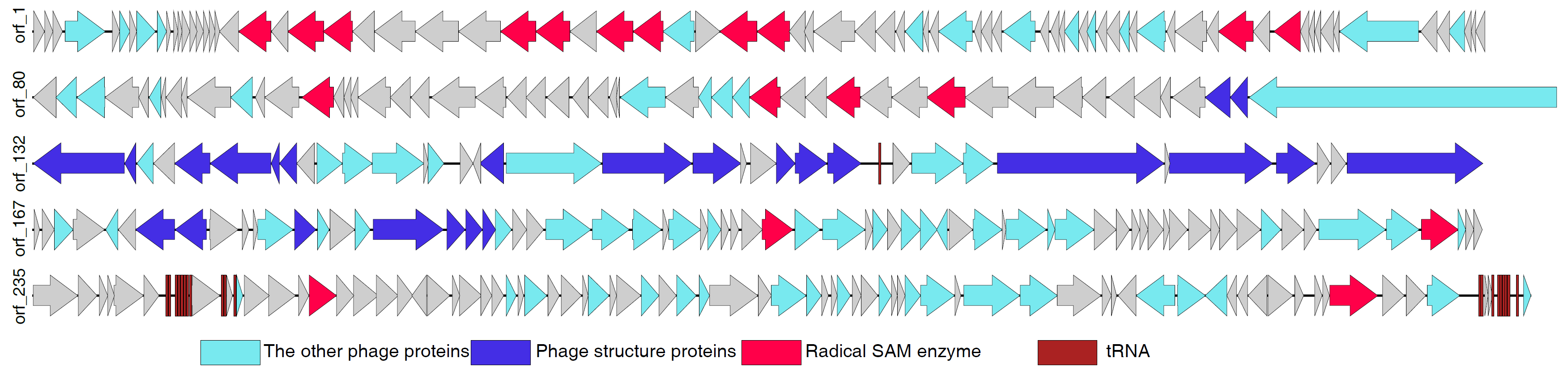


**Supplemental Fig. 9** Tertiary structures of O-antigen synthesis related putative AMGs based on structural modelling using Phyre2.


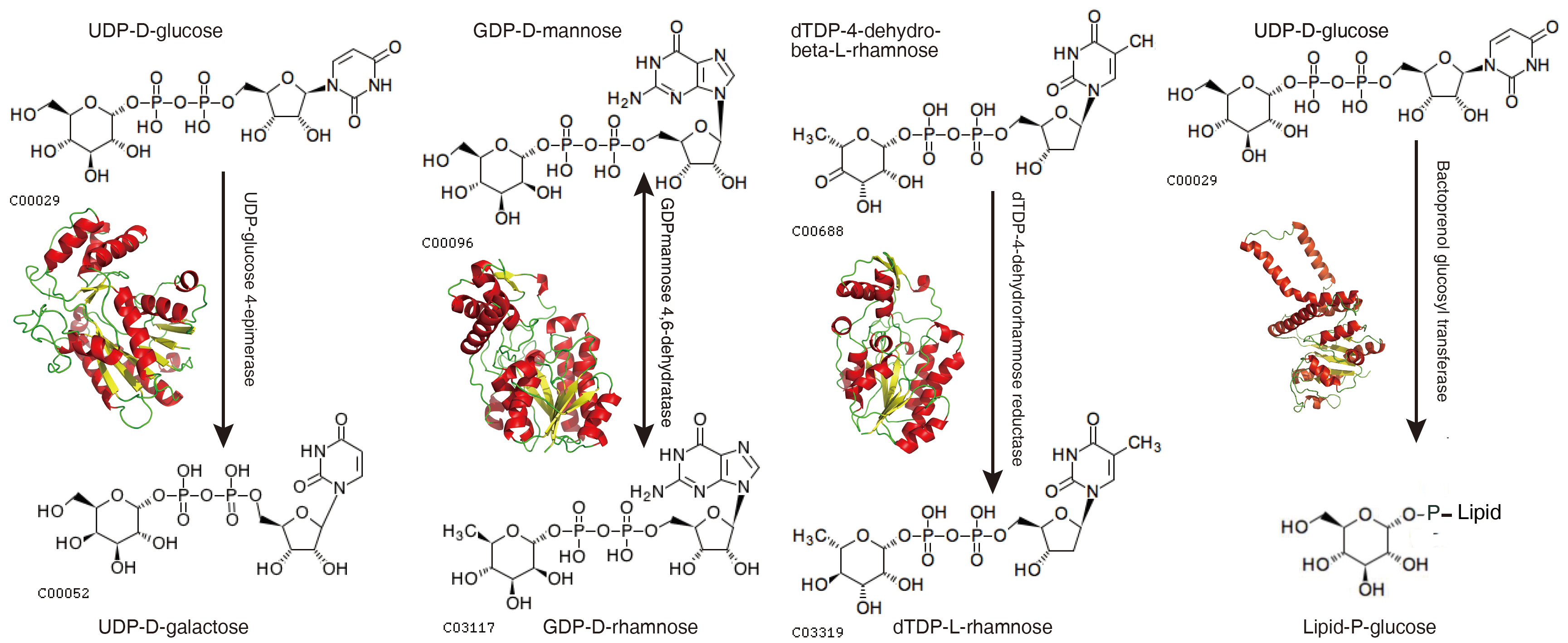

Supplement: Supplementary file 1 — Supplementary information [file 43705_2022_135_MOESM1_ESM.docx]
